# Integrated Assessment for the Estrogenic Effects of Pyrethroid Compounds: Defining the Molecular Initiating Events and Key Events for the Adverse Outcome Pathway

**DOI:** 10.3390/toxics12030218

**Published:** 2024-03-15

**Authors:** Darlene Mae D. Ortiz, Juyoung Park, Handule Lee, Kwangsik Park

**Affiliations:** College of Pharmacy, Dongduk Women’s University, Seoul 02748, Republic of Korea

**Keywords:** pyrethroids, estrogenic effects, adverse outcome pathways, IATA, ITS

## Abstract

Pyrethroids, which are derived from natural insecticides found in chrysanthemum flowers, are widely utilized in various sectors, including agriculture, forestry, horticulture, and personal insect protection. Due to their widespread use, concerns have arisen regarding their potential estrogenic effects on female reproductive health. This review aims to address data gaps and inconsistencies in previous studies by defining molecular initiating events and key events within the adverse outcome pathway associated with pyrethroid-induced estrogenic effects. To achieve this, we propose utilizing Integrated Approaches to Testing and Assessment (IATA), which incorporate in vitro assays and in vivo assessments to comprehensively investigate the estrogenic effects of pyrethroids. An initial search was conducted in the PubMed database to identify relevant articles. Subsequently, the findings were classified according to the IATA strategy. This review provides an overview of the current understanding of pyrethroids and their estrogenic effects, identifies data gaps, and highlights the use of IATA in existing studies on the estrogenic effects of various pyrethroids. It emphasizes the urgent need for comprehensive research on the estrogenic effects of pyrethroids and highlights the importance of standardized testing methods like IATA to accurately assess their impact on human and environmental health. By promoting the use of Integrated Testing Strategies (ITSs) and addressing data gaps, researchers and regulators can enhance the accuracy of assessments, ensuring better protection of human and environmental health from the potential estrogenic effects of pyrethroid exposure.

## 1. Introduction

Over the past few decades, the prevalence of pyrethroids has surged, largely attributed to the phased use of organophosphate pesticides owing to their documented harm to mammals [1]. Pyrethroids, a class of synthetic chemicals derived from natural insecticides in chrysanthemum flowers known as pyrethrins, have been extensively applied in agriculture, forestry, horticulture, and personal protection against insects, such as mosquitoes [2]. Owing to their stability and heightened potency in various environments, pyrethroids have become the preferred choice in the pest control industry because of their efficiency and prolonged effectiveness [3,4]. Classified as type I, with a basic cyclopropane carboxylic ester structure (e.g., allethrin, bifenthrin, d-phenothrin, permethrin, resmethrin, and tetramethrin), and type II, which incorporates a cyano group (e.g., cypermethrin, deltamethrin, cyhalothrin, cyfluthrin, fenvalerate, and their analogs), pyrethroids display a diverse array of chemical compositions (Table 1) [5,6,7]. Pyrethroid exposure can result in various adverse effects including impaired neurodevelopment, major chronic diseases including cardiovascular disease, carcinogenicity, and reproductive dysfunction [1]. Specifically, due to their widespread global use, concerns regarding the potential impact of pyrethroid exposure on endocrine health are escalating, exacerbated by its stability in the environment and anticipated prolonged persistence, raising the risk of widespread contamination with potential consequences for various organisms and ecosystems [7,8,9,10]. Pyrethroids have been consistently detected in residential homes, food, and human samples, highlighting the pervasive nature of exposure [9,10]. The concern is that it is not only limited to occupational workers but also to the general population who are potentially exposed to pyrethroids. Recent exposure population surveys in several countries have indicated a slight increase in environmental exposure, particularly among workers using pyrethroid insecticides, with a 1.5–2-fold rise observed in the United States [11,12].

In recent years, the escalating use of pyrethroids has raised concerns regarding their potential impacts on human reproductive health, particularly given the complex mechanisms of toxicity observed in the male and female reproductive systems. Previous studies have elucidated various facets of pyrethroid toxicity, including their interference with androgen receptors, inhibition of steroid synthesis, modulation of the hypothalamic-pituitary-gonadal axis, action as estrogen receptor modulators, and induction of oxidative stress. Notably, evidence suggests a decrease in ovarian reserve following pyrethroid exposure [8,13].

While several studies have summarized the effects of pyrethroids on male and female fertility, the existing body of knowledge leans towards male reproductive health. For instance, investigations into the relationship between synthetic pyrethroids and male reproductive hormones, particularly 3-Phenoxybenzoic acid (3-PBA), have revealed significant associations. Another study showed that elevated pyrethroid exposure was correlated with increased luteinizing hormone levels and reduced estrogen concentrations in non-occupationally exposed Chinese men [14,15]. Moreover, studies have observed an increase in follicle-stimulating hormone levels in men with higher 3-PBA pyrethroid metabolites, along with a suggestive decline in the free androgen index [16]. Other studies have reported lower sperm concentrations in parallel with increased 3-PBA levels [16,17]. However, data on the effects of pyrethroids on female fertility are comparatively sparse. Few studies have explored the relationship between pyrethroids and reproductive hormone levels in females. Notably, one study associated indoor pyrethroid use with decreased plasma anti-Müllerian hormone concentrations, indicative of potential adverse reproductive outcomes [18]. Another cohort study involving 615 Chinese women seeking to conceive found an association between increased urinary pyrethroid metabolite 3-PBA concentration, prolonged time to pregnancy (TTP), and decreased fertility [19]. The increasing use of pyrethroids, as evident in both biomonitoring and epidemiological studies, emphasizes the urgent need to address the potential negative effects of increased human exposure on reproductive health [12]. 

To assess the risk associated with pyrethroids, comprehensive risk assessments involving key components such as exposure assessment, hazard identification, dose-response assessment, and risk characterization are imperative. Recognizing the need for a systematic and informed approach, organizations such as the Organization for Economic Co-operation and Development (OECD) and the United States Environmental Protection Agency (EPA) advocate an Integrated Approach to Testing and Assessment (IATA). IATA, a methodology in toxicology and risk assessment, offers a comprehensive framework for evaluating the potential hazards and risks associated with chemical substances [20,21]. This approach can address data deficiencies regarding pyrethroids and prioritize areas for further investigation. Embracing the principles of the 3Rs (Replacement, Reduction, and Refinement) in animal testing, the IATA promotes the use of alternative methods and seeks to minimize unnecessary animal testing.

An electronic search was conducted in the National Library of Medicine (PubMed) database using the keywords “pyrethroids, estrogenic, in vitro, in vivo, as well as individual names of all pyrethroids.” A systematic review was performed on the selected articles, followed by a further filtering process to include only those that addressed the estrogenic effects of pyrethroids specifically on reproductive health. 

This study highlights several important aspects: (1) it discusses the current understanding of pyrethroids and their estrogenic effects, specifically focusing on their impact on the female reproductive system; (2) it identifies gaps in the available data; (3) it proposes the use of the IATA method for screening; and (4) it offers insights into future perspectives on pyrethroid screening methods and their potential to predict adverse outcomes. The overall goal of this review was to enhance our understanding of the endocrine-disrupting effects of pyrethroids, with a particular emphasis on their estrogenic impact, and to demonstrate the utility of the IATA in predicting adverse outcomes.

## 2. IATA for Estrogenicity

**Figure 1:**
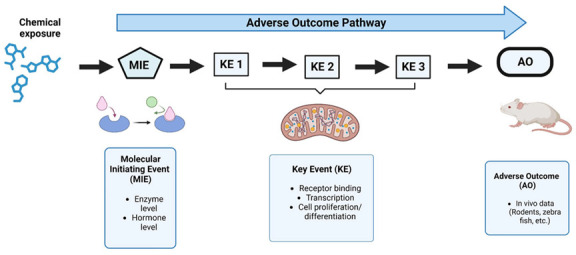
ITS for the IATA of estrogenic chemicals for endocrine-disrupting effects.

Estrogenicity, the ability of a substance to mimic the effects of estrogen in the body, is a common form of endocrine disruption that can lead to a range of adverse biological effects [22]. To measure estrogenic activity, various screening methods are presented by the IATA to evaluate the estrogenic effects of certain compounds. These methods include the incorporation of molecular initiating events (MIEs) and key events (KEs) to predict possible outcomes, which helps to minimize the need for extensive testing while enhancing the reliability of adverse outcomes. IATA tests include both in vitro and in vivo studies to assess the effects of substances on reproduction and fertility (Figure 1).

### 2.1. MIE to Assess the Estrogenic Effect of the Pyrethroids

The MIE of the endocrine system orchestrates a symphony of hormonal signals that is critical for proper functioning and development. External factors, such as exposure to harmful chemicals, can disrupt this balance, leading to endocrine disruption. The MIE sets a series of molecular responses into motion. It is the initial interaction point between an external chemical and the endocrine system, identifying possible disruptions. MIE includes changes in endocrine-related enzyme and hormone levels. 

#### 2.1.1. Enzymes for Steroidogenesis

Steroidogenesis refers to the biological process by which steroid hormones, including sex hormones, are synthesized within the body. This process relies heavily on the involvement of various enzymes that play critical roles in different steps of steroidogenesis. Dysfunction of these enzymes can disrupt the pathways in which they participate, leading to various health issues and hormonal imbalances. Although some studies do not directly measure enzymatic expression, they often determine enzyme levels by targeting genes that directly regulate the enzymes. Furthermore, genetic changes can alter enzymes coding for steroidogenesis. Human adrenocortical H295R cells have been validated by OECD Test Guideline 456 to detect chemicals that disrupt testosterone and 17β-estradiol (estradiol) biosynthesis [23]. The primary genes involved in steroidogenesis include steroidogenic acute regulatory protein (StAR), cytochrome P450 side-chain cleavage enzyme (CYP11A1), 3-beta-hydroxysteroid dehydrogenase (3-beta-HSD), cytochrome P450 17-alpha-hydroxylase/17,20-lyase (CYP17A1),17-beta-hydroxysteroid dehydrogenase (17-beta-HSD), and aromatase cytochrome P450 (CYP19) [24]. Several studies have investigated the effect of pyrethroids on the mRNA expression levels of genes involved in steroidogenesis. Zhang et al. explored the influence of cypermethrin isomers (α- β-, and θ-cypermethrin) on the steroidogenic pathway. The study found that all three stereoisomers suppressed the expression of the 3-βHSD gene. This decrease in 3-βHSD might suggest a hindrance to the conversion of cholesterol into different hormones. Additionally, both α- and β-cypermethrin significantly stimulated the expression of 17βHSD and CYP19, which are involved in estrogen synthesis. CYP19 is the only gene that encodes the aromatase enzyme required for the formation of E_2_, and such results suggest an increase in cellular estrogen levels, which may lead to various health issues including breast cancer, endometriosis, and uterine cancer [25]. Another study reported a 31.3% decrease in 3-βHSD levels in female rats after two weeks of treatment with 50 mg/kg cypermethrin [26]. Another study by Liu et al. investigated the effect of bifenthrin on steroidogenic enzymes, including StAR and CYP19. The expression of StAR, a gene associated with cholesterol metabolism, decreased in rat ovarian granulosa cells, indicating that bifenthrin may interfere with the initial step of steroid hormone production and thus affect steroidogenesis. Furthermore, CYP19a1 was significantly downregulated, suggesting a possible interference with estrogen biosynthesis. In addition, this study demonstrated that bifenthrin inhibited luteinizing-hormone-responsive ovulatory genes both in vitro and in vivo. These findings highlight the potential of bifenthrin to disrupt the reproductive function in females [27]. On the other hand, in a study conducted by Andersen et al., aromatase enzyme CYP19 activity in human placental microsomes was assessed with the use of deltamethrin. The results of their study indicated that the compound did not exhibit inhibitory effects on aromatase activity at 50 µM, which suggests that it may not interfere with the function of sex hormones [28]. 

CaBP-9k functions as a chaperone and plays a critical role in the regulation of steroidogenic enzymes. Research has shown that uterine CaBP-9k is highly responsive to exogenous estrogen (E_2_) and is a sensitive tool for the identification of estrogenic compounds [29]. In one study, co-administration of tetramethrin and E_2_ resulted in significant inhibitory effects and elevated levels of CaBP-9k mRNA at doses of 5, 200, and 800 mg/kg [30]. Treatment with permethrin (10–800 mg/kg) produced similar results, with elevated levels of CaBP-9k mRNA observed in immature rats [31]. This evidence indicated the estrogenic ability of some pyrethroids, including tetramethrin and permethrin. Current evidence suggests that pyrethroids may have estrogenic effects through changes in specific enzyme-related genes that disrupt the steroidogenesis pathway. 

#### 2.1.2. Hormone Levels

The relationships between enzymes and hormones are complex. Hormones can influence enzyme activity by binding to specific receptors on the cell surface either by activating or inhibiting enzymes. Enzymes also play a crucial role in the regulation of hormone production and secretion. Thus, these two components are intricately connected. Estrogen, a key hormone involved in estrogenicity, is produced by the ovaries and is essential for reproductive and developmental processes as well as for maintaining hormonal balance. However, excess estrogen or imbalances in estrogen-related pathways may result in adverse health effects such as reproductive disorders and disruptions in the menstrual cycle [32,33]. Progesterone, another hormone involved in estrogenicity, is produced by the ovaries, regulates the menstrual cycle, and prepares the body for pregnancy. Although testosterone is typically considered a male hormone, it can also affect estrogen levels in the body. High testosterone levels can lead to increased estrogen production [34]. When chemicals cause abnormal levels of these hormones, they directly translate into possible estrogenic effects. 

Pyrethroids were found to affect steroidogenic enzyme-related genes, and similar results were observed for hormones. Few studies have directly investigated the effects of pyrethroids on hormone levels. A previous study provided important epidemiological evidence for the association between pyrethroids and sex hormones following exposure to pyrethroids in both male and female adults. Studies have found that pyrethroid exposure is positively associated with total testosterone (TT) and sex-hormone-binding globulin (SHBG). Additionally, pyrethroid exposure is negatively associated with circulating free testosterone levels in males. Overall, these findings suggest that pyrethroids may interfere with the human endocrine system [35]. Additionally, an in vivo study on lambda-cyhalothrin, a type II pyrethroid, showed decreased levels of estradiol and progesterone hormones, along with estrogen-related genes, after treatment (6.3 mg/kg BW and 11.33 mg/kg) for 14 days. Moreover, this study revealed that lambda-cyhalothrin exposure was associated with decreased body weight and food intake as well as increased liver and kidney toxicity. These findings suggest that lambda-cyhalothrin causes endocrine and reproductive disruptions [36]. Few studies have directly measured hormone levels after exposure to pyrethroids. Therefore, further research is necessary to thoroughly assess the endocrine-disrupting effects of pyrethroids, in line with current investigations (Table 2). 

### 2.2. Key Events (KE) to Assess the Estrogenic Effect of the Pyrethroids

Following MIE, key events were used to assess estrogenicity to evaluate the potential impact of pyrethroids. Various key events and assays have been employed to assess estrogenicity, providing valuable insights into the potential health risks associated with exposure. The current key events from the OECD suggested assays and methodologies such as estrogen receptor binding, transactivation of estrogen receptors, and cell proliferation and differentiation to assess the estrogenicity of chemicals.

#### 2.2.1. Estrogen Receptor Binding 

Pyrethroids have been investigated for their ability to interact with estrogen receptors (ERα and ERβ), as aberrant estrogenic signaling has been associated with endocrine-related disorders [38]. While other studies have shown that pyrethroids do not directly bind to estrogen receptors, they can mimic the effects of estrogen in the body because of their ability to bind to other receptors that are involved in the regulation of estrogen levels. They can bind and activate estrogen receptors, showing a response similar to estrogen, which may interfere with the synthesis, metabolism, binding, and cellular responses of natural estrogens [37]. The interaction of pyrethroids with the estrogenic pathway in vitro varies depending on the assay and cells employed, ranging from one compound to another. Chen et al. performed an estrogen receptor competitive binding assay of pyrethroids. Initially, radio-inert 17β-estradiol was tested, and it effectively competed with [3H]-E_2_ for binding to ER at the concentrations tested (10^−4^–10^−12^ M). Among the four pyrethroids tested (fenvalerate, cypermethrin, permethrin, and deltamethrin), fenvalerate and cypermethrin were the most effective in inhibiting the binding of [3H]-E_2_ to the ER. The IC_50_ values were 0.479 and 0.562 mM, respectively, while permethrin and deltamethrin inhibited the binding of [3H]-E2 to the ER, which was less than 67%. This emphasizes that both fenvalerate and cypermethrin strongly block the binding of E_2_ to its receptors, whereas deltamethrin and permethrin still bind to ER receptors, but to a lesser degree and resulted in interference by estrogen [39]. In another study, Saito et al. investigated the estrogenic effects of d-trans-allethrin, cypermethrin, empenthrin, fenvalerate, imiprothrin, permethrin, d-phenothrin, and prallethrin through competitive ligand-binding assays. Using a dose-dependent displacement of fluoromone ES1 from hERα, results show, however, that no apparent effects were observed, suggesting that all the pyrethroids tested have no potential for receptor binding under the experimental conditions employed [40]. Another study demonstrated the estrogenic and anti-estrogenic activities of seven pyrethroid insecticides (bioallethrin, cypermethrin, fenvalerate, permethrin, sumithrin, and tetramethrin) using competitive estrogen receptor (ER) binding assays. The results showed that none of the pyrethroid insecticides competitively inhibited the binding of [3H]-E_2_ to rat uterine ERs [37]. Another study evaluated the ERα and ERβ competitive binding ability of permethrin, cypermethrin, and fenvalerate. However, none of the pyrethroids tested displayed the ability to inhibit the binding of E2 to ERα and Erβ [41]. The contrasting results from different studies might be due to the responses of different test assays used, which limits the reliability of the conclusion regarding the estrogenicity of pyrethroids (Table 3). 

#### 2.2.2. Transactivation of the Estrogen Receptor

In contrast to ER binding, transactivation is a more complex process that involves the recruitment of additional factors. The main difference between the two is that ER binding requires the presence of estrogen, whereas ER transcription can activate gene expression in the absence of estrogen, which occurs when ER binds to estrogen response elements (EREs) in the promoter regions of target genes. This leads to the recruitment of transcriptional coactivators and the activation of gene transcription [42]. Extensive research has been conducted on the pS2 gene, which is regulated by estrogen and thought to contribute to the cellular response to this hormone. In particular, pS2 is believed to play a role in the transcriptional regulation of estrogen receptor alpha (ERα), the primary receptor for estrogen in target cells. Furthermore, pS2 plays a crucial role in regulating the growth and survival of breast cancer cells, and mutations in this gene have been linked to the development and progression of breast cancer [43]. MCF7 cells, a human breast cancer cell line, are often used to assess estrogen-responsive genes, such as pS2, which has been shown to promote cell proliferation and inhibit apoptosis. In studies on the estrogenic effects of pyrethroids, the pS2 gene has been evaluated, with some pyrethroids showing a significant increase in pS2 mRNA expression after exposure to chemicals [44]. For example, sumithrin showed a significant increase in pS2 mRNA at 10^−5^ M after three days of exposure in MCF7 cells. Additionally, elevated pS2 expression was observed after treatment with 0.001 μM cypermethrin and permethrin. Moreover, a significant increase in ERα has been correlated with increased pS2 levels [37]. By modulating the expression of ERα, pS2 can influence the responsiveness of cells to estrogen and the subsequent signaling pathways activated in response to estrogen. 

Studies have shown that pyrethroids regulate ER transcript expression. Moreover, after either stable or transient transfection with one or two isoforms of human ER (e.g., human HeLa cells), some pyrethroids showed either negative or weak responses with no consistent pattern. Garey and Wolff evaluated four pyrethroids (fenvalerate, sumithrin, d-trans allethrin, and permethrin) as estrogen agonists and antagonists in the Ishikawa Var-I human endometrial cancer cell line and T47D human breast cancer cell line. Fenvalerate and sumithrin demonstrated significant estrogenicity at 10 µM. However, none of these compounds showed statistically significant estrogen antagonist activity [45]. Taylor et al. determined the estrogen receptor transcripts of pyrethroids using a yeast assay. Results showed that pure cypermethrin (36 µM) and permethrin metabolite (0.69–69 nM) induce near-maximal estrogen-receptor-mediated β-galactosidase expression showing a clear estrogenic response. Similarly, cypermethrin obtained from a commercial garden product induced a more potent estrogenic response in the yeast assay (0.36 nM). Moreover, exposure to cypermethrin significantly increased the expression of estrogen receptors in mouse Sertoli cells. This effect of pyrethroid on estrogen receptor gene transactivation suggests a disruption of normal estrogen signaling [38,46]. Another study by Saito et al. showed no response in a mammalian cell-based luciferase reporter gene assay with pyrethroids, even at high concentrations (10 µM). This suggests lack of influence on the transactivation of hERα-regulated genes by classic hERα-mediated mechanisms in pyrethroid insecticides [40]. In another study, no response was detected at the maximum concentration (5 × 10^−5^ M) of deltamethrin in the ER transactivation assay using MCF7 cells [28]. Another study utilized the chemical-activated luciferase gene expression (CALUX) assay using the human ovarian carcinoma (BG-1) cell line to assess the concentration-dependent agonist and antagonist effects of permethrin and bifenthrin on estrogen receptors. However, neither pyrethroid was able to agonize with ER at all concentrations tested (1–1000 µg/L). In contrast, bifenthrin (1–100 ng/L bifenthrin) displayed a concentration-dependent decrease in the ability of E_2_ to induce ER-dependent reporter gene activity in the ER antagonism assay. This was observed with the recovery of estrogenic activity at concentrations greater than 100 ng/L. Permethrin, on the other hand, displayed a 30–40% reduction in maximal E_2_ activity, but no concentration dependence was observed. Additionally, no transactivation was observed after exposure of HeLa cells to cypermethrin, fenvalerate, and permethrin [47]. 

Another study by Du et al. determined the estrogenic activities of pyrethroids, including cycloprothrin, cyfluthrin, cyhalothrin, cypermethrin, deltamethrin, etofenprox, fenvalerate, permethrin, and tetramethrin. Out of the pyrethroids tested, four (cyhalothrin, deltamethrin, fenvalerate, and permethrin) induced minimal luciferase activity, exhibiting a very weak estrogenic activity at 10^−5^ M in the ER-mediated reporter gene assay using CV-1 cells. Furthermore, four of the pyrethroids tested, including cycloprothrin, etofenprox, cyfluthrin, and permethrin, displayed antiestrogenic effects in competition with 1 × 10^−9^ M of E_2_. This study concluded not only a weak estrogenic effect for some pyrethroids but also antiestrogenic activities [48]. Although there are a few studies on the transactivation of pyrethroids, contrasting results have been reported in previous studies. For example, fenvalerate was concluded to be negative in a study by Saito et al., in which HeLa cells transfected with expression vectors (pRc/RSV-hER and pGL3-TATA-ERE X 5) were utilized with a maximum dose of 10 µM [40]. However, Lemaire et al. also used HeLa cells transfected with GAL4RE-ERE-βGlob-Luc-SVNeo plasmid, resulting in 55% transactivation activity of ERα. In contrast, other pyrethroids showed no significant activity at similar doses (permethrin, cypermethrin) [41]. Similarly, contrasting studies on cyhalothrin were also observed, where it induced minimal luciferase expression and showed little estrogenic activity at 10^−5^ M in CV-1 cells, while another study showed no estrogenic response in CHO-K1 cells. However, antiestrogenic properties were observed in the latter study, inhibiting E_2_ at 10^−11^ M while the former did not show any antiestrogenic response [41,48]. This highlights the data gaps and the employment of different assay systems when evaluating the estrogenic effects of pyrethroids, as different assay systems were used to assess the estrogenic and antiestrogenic activities of pyrethroids. Thus, both these issues should be addressed to provide more accurate and precise data on pyrethroids (Table 4).

#### 2.2.3. Cell Proliferation and Differentiation 

Cell proliferation and differentiation are two important biological processes essential for the growth and development of living organisms. Cell proliferation refers to the process of cell division, which results in the production of new cells. This process is essential for growth and repair of tissues and organs. On the other hand, cell differentiation refers to the process by which cells become specialized to perform specific functions. This process is important for the development of complex organisms and for maintaining the proper functioning of tissues and organs [49]. Pyrethroids have been shown to have cytotoxic effects on certain cell types, and their use in agriculture and other settings has raised concerns regarding their potential toxicity to non-target organisms. Therefore, studying the effects of pyrethroids on cell proliferation and differentiation can provide important information regarding their potential risks and benefits. Furthermore, the effect of pyrethroids on cell proliferation is a complex and context-dependent phenomenon that can vary based on the specific type of pyrethroid, cell type, concentration, and exposure duration. Owing to their potential to disrupt endocrine function, pyrethroids have been the focus of studies using hormonally responsive biological assays. An additional method to further confirm and characterize the estrogenic properties of chemicals involves the expression of estrogen receptors (ER) via the proliferation of the MCF-7 human breast cancer cell line. This cell line is widely recognized as an in vitro system and is characterized by its sensitivity to ER [50]. In MCF-7 cells, pyrethroids can disrupt the balance between proliferation and differentiation, leading to abnormal cell growth and development. Pyrethroids have the potential to act as xenoestrogens, thus mimicking the effects of estrogen in the body and possibly leading to reproductive development dysfunction. Go et al. evaluated the estrogenic properties of sumithrin, fenvalerate, d-trans allethrin, and permethrin, and their results showed that sumithrin and fenvalerate induced significant levels of cell proliferation in a dose-dependent manner, starting from 10 pM and 10 nM, respectively. D-trans allethrin slightly induced proliferation at 10 ρM, whereas permethrin had a noticeable effect on cell proliferation at 100 pM. Therefore, both sumithrin and fenvalerate have significant estrogenic properties and can induce cell proliferation, whereas permethrin and d-trans allethrin have minimal or no impact on these cellular processes [51]. Another study showed a dose-dependent cell proliferation effect of sumithrin, with the highest induction observed at 10 ^−5^ M. The level of sumithrin induction reached 137% of the control, with a maximum proliferation equivalent to 70% of that of 17β-estradiol (E_2_), indicating weak estrogenic activity [37]. Another study revealed the proliferative effects of fenvalerate (10^−8^ M), permethrin (10^−8^ M), deltamethrin (10^−7^ M), and cypermethrin (10^−9^ M) on cell proliferation, demonstrating a significant increase compared to the control group, with proliferation fold values of 2.17, 2.69, 1.74, and 1.46, respectively. These four pyrethroids displayed a partial agonistic response, in contrast to the proliferative effect of E2 (3.46-fold). Notably, this study used the ER antagonist ICI 182.780 to determine whether pesticide-induced proliferation was due to ER binding. The results showed that the proliferation induced by cypermethrin, permethrin, and deltamethrin was inhibited by ICI 182.780, whereas fenvalerate was only partially blocked. 

These findings further support the involvement of the ER in mediating pyrethroid pesticide-induced proliferation [39]. Assessment of fenvalerate in two different cell carcinoma cell lines suggested differential effects on proliferation. Increasing the concentration of fenvalerate exposure (0.01–100 μM) significantly promoted the proliferation of MCF-7 cells. In the MDA-MB-231 cell line, however, proliferation was suppressed for a longer time, and toxicity was observed at higher concentrations [52]. Another cypermethrin study showed a significant increase in proliferation, reflecting cellular metabolic activity and energy (ATP) of 125% at 0.1 μM concentration. Additionally, co-exposure to E2 (0.1 nM) reached 373% of the total ATP level in the control. Permethrin exposure, on the other hand, resulted in a 116% ATP compared to the control with a 364% ATP increase with estradiol co-exposure compared to the control. Both pyrethroids have been extensively studied owing to their potential estrogenic effects. This result supports their ability to exhibit estrogenic activity through a significant effect on cell proliferation [53]. Another permethrin study of proliferation resulted in increased cell viability in a dose-dependent manner from 10^−7^ M to 10^−4^ M for 72 h of incubation with MCF-7 cells [54]. Another extensively studied pyrethroid, lambda-cyhalothrin (LCT), showed a dose-dependent increase in cell proliferation. Specifically, a 10^–7^ M dose of LCT caused a 2.0-fold increase in cell proliferation, with a relative proliferative effect of 45%. To confirm its binding with ER, inhibition of the estrogen receptor (ER)-antagonist completely blocked the cell proliferation effect of LCT [55]. Some studies have also measured not only pyrethroid compounds but also their enantiomers. Enantiomers have the same chemical formula but can exhibit different biological activities, including estrogenicity [56]. Among these is the evaluation of the enantiomers of bifenthrin, which is another pyrethroid compound. 1S-cis-BF (10^−9^ M) and 1R-cis-BF (10^−8^ M) showed relative proliferative effects (RPE) of 74% and 20.9%, respectively. Moreover, the addition of the estrogen receptor antagonist ICI 182,780 resulted in an inhibitory effect, suggesting that proliferation was indeed mediated via the estrogen receptor pathway [57]. Another study investigated the presence of permethrin and beta-cypermethrin, along with the metabolites phenoxybenzoic alcohol (PBCOH), 3-phenoxybenzaldehyde (PBCHO), and 3-phenoxybenzoic acid (PBCOOH). The findings indicated a concentration-dependent increase in cell proliferation with permethrin and cypermethrin, with the most notable rise at 10^−7^ M comparable to E2 at 10^−9^ M, and relative proliferative effect ratios of 55.4% and 56.3%, respectively. Additionally, similar results were observed for the metabolites PBCOH and PBCOOH, suggesting that they also exhibit estrogenic activities, as they were able to stimulate the growth of estrogen-responsive MCF-7 cells in culture at doses ranging from 10^−7^ to 10^−6^ M. However, PBCHO was not capable of inducing cell proliferation [32]. This study emphasizes the importance of considering not only the estrogenic effects of the parent compounds but also their metabolites when assessing their potential impact. Nonetheless, the overall data on cell proliferation support the conclusion that pyrethroids exhibit estrogenic activity (Table 5).

When cells differentiate, they alter their shape, size, and energy requirements. Certain chemicals disrupt normal cell function, causing more cells to oxidize and initiate a chain reaction that ultimately inhibits signaling pathways [58,59]. Pyrethroids can disrupt cellular processes and affect cell differentiation. Similar to other xenoestrogens, pyrethroids can interfere with the activation of the transcription factors necessary for the differentiation of specific cell types. In one study, pre-treatment of mesenchymal stem cells (MSC) with 100 μM of permethrin and cypermethrin significantly increased the adipogenic differentiation ability of cells, as suggested by lipid accumulation and increased expression of the adipogenic markers PPARγ, C/EBPα, and FABP4. Moreover, both pyrethroids mediated autophagy at 24 h. The results indicated that pyrethroid treatment at a specified concentration positively affected the adipogenic differentiation of MSCs [60]. Another study on pyrethroid cell differentiation assessed the immunotoxic effects of β-cypermethrin and 3-PBA on human promyelocytic leukemia cells (HL-60). These pyrethroid compounds have been found to inhibit the granulocytic differentiation of HL-60 cells. This suggests that exposure to pyrethroids may reduce the population of granulocytes in an organism, potentially increasing its susceptibility to pathogens. In addition, β- cypermethrin and 3-PBA also inhibited the mRNA expression of transcription factors PU.1 and C/EBPε, which play critical roles in promoting granulocytic differentiation. This indicates that pyrethroids may impede the differentiation of immune progenitor cells, potentially affecting the ability of the immune system to combat infections [61]. 

Previous studies have suggested that pyrethroids may affect cell differentiation. Although current evidence does not clearly establish a link between pyrethroid exposure and estrogenic effects on differentiation or the endocrine system, this implies that such exposure could result in cell differentiation that may potentially influence the estrogen-mediated pathway. Further research in this area is necessary to investigate this possibility. Overall, the aforementioned studies have highlighted the potential impact of pyrethroids on both cell proliferation and differentiation, highlighting the need for further investigations to identify any associated risks.

## 3. Adverse Outcome of Estrogenicity

Estrogenicity is a well-known factor that can cause significant disruptions to the endocrine system, which can ultimately lead to reproductive issues in both males and females, a serious adverse outcome. In vivo models are necessary to predict these adverse effects because they more accurately represent how a substance affects living organisms than in vitro models. Moreover, although in vitro testing is a valuable tool for screening and mechanistic studies, they have limitations as to predicting in vivo responses accurately. In vivo models are useful for studying the effects of substances on organisms, including their interactions with multiple organs and systems. In vivo data were also employed as endpoints to evaluate the adverse effects of the chemicals, such as their estrogenic effects. Thus, it is essential to complement in vitro data with in vivo studies to better understand the potential effects of substances on living organisms [62,63]. 

One such bioassay used in in vivo data gathering is the uterotrophic assay, a short-term screening test that measures the increase in uterine weight or uterotrophic response in either ovariectomized adult females or immature non-ovariectomized animals, typically rodents. This assay is included in the “OECD Conceptual Framework for the Testing and Assessment of Endocrine Disrupting Chemicals” at Level 3 as an in vivo assay that provides data on a single endocrine mechanism such as estrogenicity [64]. 

Uterotrophic activity refers to the capacity of a substance to promote uterine contractions and potentially affect the endometrium. The examination of pyrethroids through in vitro and in vivo testing has revealed contrasting results, emphasizing the limitations of relying solely on in vitro screening for predicting the estrogenic effects of certain pyrethroids. For instance, permethrin has demonstrated negative estrogenic effects in ER receptor binding, transcription, and cell proliferation, suggesting its potential to disrupt estrogenic activity. However, the uterotrophic assay revealed a positive estrogenicity, indicating that in vitro screening alone may not provide a comprehensive assessment of a pyrethroid’s estrogenic potential. Similarly, tetramethrin, which showed no estrogenicity in in vitro screening tests (ER receptor binding, transcription, and cell proliferation), exhibited estrogenicity in vivo screening. Thus, the reliance on in vitro screening tests is insufficient in accurately predicting the estrogenic effects of certain pyrethroids. This signifies the importance of conducting in vivo screening tests to assess the estrogenicity of pyrethroids.

Furthermore, it is essential to recognize that variations in uterotrophic activity can lead to adverse impacts on fertility. Studies have demonstrated that exposure to substances exhibiting estrogenic properties, such as pyrethroids, can result in increased uterine weight and structural changes. Therefore, it is crucial to accurately assess the estrogenic potential of pyrethroids to ensure their safe use and minimize potential risks to reproductive health.

### 3.1. Uterine Weight 

Evaluating estrogenic compounds often involves uterine weight assessment, which involves the removal of the uterus from rodents and subsequent weighing. This measurement is a crucial indicator of potential toxicity. In ovariectomized rats, uterotrophic evaluation by Kunimatsu et al. showed no increase in uterine weight after oral exposure to esfenvalerate (5, 10, or 20 mg/kg/day), fenvalerate (20, 40, or 80 mg/kg/day), or permethrin (37.5, 75, or 150 mg/kg/day) for 3 days. Reference chemicals consisting of ethynyl estradiol (0.03 mg/kg/day) and methoxychlor (125 mg/kg/day) both showed a significant effect in this assay protocol, thus indicating that none of the pyrethroid tested showed estrogenic effects at dose levels below those causing excessive systemic toxicity [65]. Similarly, in another study, evaluation with fenvalerate showed no significant increase in relative uterine weight compared to the vehicle group at varying doses (0.4, 1, 4, 8, and 40 mg/kg/day) for 3 consecutive days. The positive control (estradiol benzoate), however, significantly increased uterine weight compared to the vehicle-treated group [66]. Another study on fenvalerate evaluated both uterine and ovarian weights of pregnant Wistar rats during gestation and lactation. Oral fenvalerate (40 mg/kg) was administered from gestational day 12 until the end of lactation. However, no significant differences were observed between the control and treated groups in terms of estrogenicity [67]. Deltamethrin, a widely used synthetic pyrethroid insecticide, is effective in controlling a range of pests. Although primarily recognized for its neurotoxic effects on insects, there is growing interest in its potential to interact with the endocrine system, specifically regarding estrogenicity. Assessment of deltamethrin for both technical and formulated compounds at doses of 2 and 4 mg/kg/day in immature female rats showed no changes in the relative uterine weight compared to the vehicle-treated group [68]. A separate investigation yielded comparable outcomes, demonstrating that exposure to deltamethrin did not result in in vivo estrogenic activity. Moreover, when subjects were simultaneously exposed to both deltamethrin and endosulfan, no estrogen-like effects were observed [69] In ovariectomized rats, uterotrophic evaluation by Kunimatsu et al. showed no increase in uterine weight after oral exposure to esfenvalerate (5, 10, or 20 mg/kg/day), following subcutaneous treatment for three days. This finding suggested the presence of estrogenic activity at doses of 800 and 200 mg/kg. Additionally, this estrogenic activity was further validated through the inhibition of permethrin-induced uterine weight by co-administration of ICI 182,780, an antiestrogen [65].

Another study has evaluated the effects of tetramethrin on living organisms. The results indicated that female Sprague–Dawley rats were administered tetramethrin at various doses ranging from 5 to 800 mg/kg/day for three consecutive days. The findings indicated a statistically significant decrease in both the absolute and relative uterine wet weights at all doses tested, implying the antiestrogenic action of tetramethrin. Additionally, tetramethrin impedes the impact of 17β-estradiol (E2) on uterine weight [30]. The use of bifenthrin, another pyrethroid, resulted in a significant increase in the mean relative wet weight and relative blotted weight of the uterus in female rats, amounting to 13.23 mg/kg of body weight after three days of treatment. Additionally, an increase in the height of uterine epithelial cells was observed in rats [70]. The assessment of the estrogenic properties of pyrethroid insecticides has produced varying results even in in vivo data. While some experiments showed no substantial changes in uterine weight or evidence of estrogenic effects from pyrethroid exposure, others found contrasting outcomes that suggested the possible estrogenic or antiestrogenic effects of specific compounds. These findings underline the complex and context-dependent nature of the pyrethroid-induced estrogenic effect and highlight the importance of considering various factors, such as dosage, duration of exposure, and experimental models, in assessing estrogenic effects. Available evidence suggests the possibility of certain pyrethroids causing toxicity; however, further research is necessary to reach definitive conclusions owing to the limited information available, particularly regarding other pyrethroids (Table 6).

### 3.2. Histological Changes and Fertility 

The intricate balance of reproductive hormones plays a pivotal role in regulating various physiological processes that are essential for fertility and fecundity. Exposure to environmental pollutants, including pyrethroids, has raised concerns about their potential to disrupt this delicate hormonal equilibrium. While pyrethroids have been primarily recognized for their neurotoxic effects, emerging evidence suggests their ability to influence reproductive hormone levels and subsequent histological changes in reproductive organs, thereby impacting fecundity. Understanding these histological alterations and their correlation with changes in reproductive hormones is crucial to elucidate the potential reproductive risks associated with pyrethroid exposure. For example, fenvalerate did not suggest any estrogenic activity following the uterotrophic assay; however, a decrease in ovarian weight was identified, signifying a potential disruption in ovarian development and function. This might suggest adverse effects on ovarian development, potentially impairing normal reproductive function [65,67]. In addition, histopathological changes were observed in the ovaries of female SD rats after oral exposure to fenvalerate for four weeks (0.00, 1.91, 9.55, and 31.80 mg/kg). According to a study conducted by He et al., damage to ovarian corpus luteum cells interferes with calcium homeostasis and causes dysfunction in the reproductive and endocrine systems [74]. Furthermore, treatment with fenvalerate resulted in a reduction in the number of ovarian follicles and vesicular atrophy of endometrial glands in both non-pregnant and pregnant rats [73]. This evidence not only accentuates the histological changes and alterations in the reproductive organs of female rodents but also highlights the potential for dysfunction in the reproductive process, which could lead to infertility.

According to a study on the toxicological effects of cypermethrin in female albino rats, a significant change in body weight was observed, accompanied by a decrease in the weight of the liver and spleen. In contrast, the kidneys showed an increase compared to the control rats. Additionally, after two and four weeks of oral cypermethrin treatment (5 and 20 mg/kg/day), an increase in the thyroid and adrenal glands was also observed. While these findings do not specifically indicate estrogenicity, they suggest that cypermethrin has toxic effects and may cause endocrine disruption [72]. Moreover, repeated treatment with cypermethrin for 30 days (5 and 20 mg/kg/day) showed significant adverse effects on the reproductive organs of both male and female albino rats. In females, cypermethrin resulted in the complete loss of follicular cells, oocytes, and albuminous fluid in the Graafian follicle. These findings suggest hazardous effects on reproductive processes and fertility in female rats [71]. Studies on deltamethrin indicate that it displays minimal estrogenic activity in living organisms. However, earlier studies have documented various histological alterations and reproductive effects associated with deltamethrin exposure. These effects include a significant reduction in implantation sites, the presence of vacuolated trophoblasts, increased leukocyte infiltration, heightened vascularization, and the presence of blood in the uterine lumen. These reproductive effects impair the interaction between the blastocyst and endometrium as well as the implantation process in female rats [75]. Additionally, low-dose exposure to deltamethrin (4.0 mg/kg) for 21 days during lactation in female rats has been shown to cause subtle changes in the reproductive behavior and physiology of their offspring at doses that did not cause maternal toxicity [78]. Another study investigated the effects of deltamethrin on the reproductive system of female Wistar rats (2.5, 5, 10 mg/kg). The results indicated significant decreases in the number of primary and secondary follicles and corpus luteum, and significant increases in atresia follicles, suggesting potential detrimental effects on the ovary tissue [77]. Similarly, reduced fertility was also observed in rats after exposure to sublethal doses of deltamethrin, followed by histopathological alterations in different organs (kidney, liver, and lungs) [76]. Exposure to pyrethroid insecticides such as fenvalerate, cypermethrin, and deltamethrin has been associated with histological alterations in reproductive organs and adverse reproductive outcomes in animal studies. Despite the lack of estrogenic effects in certain assays, pyrethroids can disrupt reproductive hormone levels and induce significant changes in the tissues and organs that are crucial for fertility.

These findings highlight the potential reproductive risks associated with pyrethroid exposure and emphasize the importance of further research to elucidate their mechanisms of action and mitigate the potential adverse effects on reproductive health.

## 4. Summary and Limitation

The current review aimed to investigate the estrogenic effects of pyrethroids in light of the limited research conducted on their disruption, particularly within the female reproductive system. To accomplish this, we collected and presented virtually all existing studies on the estrogenic effects of various pyrethroids, using the IATA screening method to identify pertinent data. This method involves MIE, which encompasses the determination of enzymatic and hormonal changes, and KEs, such as ER binding, transactivation of ER receptors, cell proliferation, and differentiation, as shown in Table 3, Table 4 and Table 5. Additionally, we present the current in vivo data to assess the adverse consequences of pyrethroids as the endpoint for evaluating the in vitro results (Table 6). This method aims to predict the potential adverse outcomes of pyrethroids in future studies without relying on in vivo data, which is the primary objective of this review. The results of in vitro experiments have demonstrated promising results, suggesting positive effects up to the in vivo stage, which supports the estrogenicity of pyrethroids. However, no definitive conclusions could be drawn at this time owing to inconsistencies in the available data. Furthermore, inconsistencies in the results from various experimenters raise concerns about the scarcity of precise end-point data. Further studies are necessary to substantiate the estrogenic properties of pyrethroids and to assess any potential health risks associated with their use. Moreover, pyrethroid pesticides have undergone extensive testing in various regulatory studies, the outcomes of which are typically confidential but are sometimes disclosed by regulatory agencies. This highlights a significant disparity between “academic” and “regulatory” science. Therefore, future investigations should take these findings into account and compare them with both in vitro and in vivo data. The present review highlights the urgent need for comprehensive research on the estrogenic impact of pyrethroids, particularly on the female reproductive system. Despite promising in vitro data supporting the estrogenic properties of pyrethroids, there are still discrepancies in the data and a lack of standardized testing methods. These data gaps and inconsistencies emphasize the urgency of implementing a standardized testing method, such as the Integrated Approaches to Testing and Assessment (IATA) recommended by the OECD, to accurately assess the estrogenic effects of pyrethroids. Furthermore, regulatory studies on pyrethroids should be addressed to bridge the gap between academic and regulatory studies.

## 5. Conclusions and Future Perspectives

Conversely, the present review offers a comprehensive overview of the existing data and the current status of pyrethroids on testing and evaluation following the OECD-based guidelines. The absence of data on several pyrethroids emphasizes the need for further research to accurately evaluate their potential risks; however, an Integrated Testing Strategy (ITS) can facilitate the detection of data gaps and predict possible adverse outcomes. This strategy involves conducting risk assessments based on the combination of exposure and assessment data. It also helps to identify potential risks and prioritize them for further investigation. Additionally, Table 7 provides a comprehensive summary of the estrogenic ITS for various pyrethroids, highlighting the significance of data-rich compounds such as cypermethrin and lambda-cyhalothrin, which demonstrate notable estrogenic effects. 

Overall, by promoting the use of ITS and addressing data gaps, researchers and regulators can enhance the accuracy and reliability of assessments, ultimately ensuring better protection of human and environmental health from the potential estrogenic effects of pyrethroid exposure.

## Figures and Tables

**Table 1:** Classification of Type 1 and Type II pyrethroids, structures, and identifications.

Classification	Pyrethroids	Structure	Molecular Formula	IUPAC Name	Cas no.	Molecular Weight (g/mol)
Type I	Allethrin	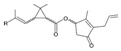	C_19_H_26_O_3_	Cyclopropanecarboxylic acid, 2,2-dimethyl-3-(2-methyl-1-propenyl)-, 2-methyl-4-oxo-3-(2-propenyl)-2-cyclopenten-1-yl ester	584-79-2	302. 4
	Bifenthrin	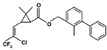	C_23_H_22_ClF_3_O_2_	(2-methyl-3-phenylphenyl)methyl (1R,3R)-3-[(Z)-2-chloro-3,3,3-trifluoroprop-1-enyl]-2,2-dimethylcyclopropane-1-carboxylate	82657-04-3	422.9
	Permethrin	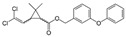	C_21_Cl_2_H_20_O_3_	(3-phenoxyphenyl)methyl 3-(2,2-dichloroethenyl)-2,2-dimethylcyclopropane-1-carboxylate	52645-53-1	391.3
	Phenothrin/Sumithrin	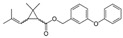	C_23_H_26_O_3_	(3-phenoxyphenyl)methyl 2,2-dimethyl-3-(2-methylprop-1-enyl)cyclopropane-1-carboxylate	26002-80-2	350.4
	Resmethrin	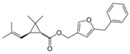	C_22_H_26_O_3_	(5-benzylfuran-3-yl)methyl 2,2-dimethyl-3-(2-methylprop-1-enyl)cyclopropane-1-carboxylate	10453-86-8	338.4
	Tefluthrin	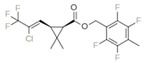	C_17_H_14_ClF_7_O_2_	rac-(2,3,5,6-Tetrafluoro-4-methylphenyl)methyl (1R,3R)-2,2-dimethyl-3-[(1Z)-2-chloro-3,3,3-trifluoroprop-1-en-1-yl]cyclopropane-1-carboxylate	79538-32-2	418.74
	Tetramethrin	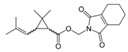	C_19_H_25_NO_4_	(1,3-Dioxo-4,5,6,7-tetrahydroisoindol-2-yl)methyl 2,2-dimethyl-3-(2-methylprop-1-enyl)cyclopropane-1-carboxylate	7696-12-0	331.406
	Metofluthrin	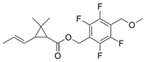	C_18_H_20_F_4_O_3_	2,3,5,6-Tetrafluoro-4-(methoxymethyl)benzyl 2,2-dimethyl-3-(prop-1-en-1-yl)cyclopropane carboxylate	240494-70-6	360.349
	Transfluthrin	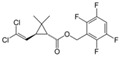	C_15_H_12_Cl_2_F_4_O_2_	(2,3,5,6-Tetrafluorophenyl)methyl (1R,3S)-3-(2,2-dichloroethen-1-yl)-2,2-dimethylcyclopropane-1-carboxylate	118712-89-3	371.15
	Prallethrin	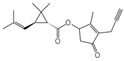	C_19_H_2_4O_3_	2-methyl-4-oxo-3-prop-2-yn-1-ylcyclopent-2-en-1-yl-2,2-dimethyl-3-(2-methylprop-1-en-1-yl)cyclopropanecarboxylate	23031-39-9	300.40
Type II	Cypermethrin	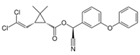	C_22_H_19_Cl_2_NO_3_	[Cyano-(3-phenoxyphenyl)methyl]3-(2,2-dichloroethenyl)-2,2-dimethylcyclopropane-1-carboxylate	52315-07-8	416.30
	Deltamethrin	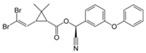	C_22_H_19_Br_2_NO_3_	(*S*)-Cyano(3-phenoxyphenyl)methyl (1*R*,3*R*)-3-(2,2-dibromoethen-1-yl)-2,2-dimethylcyclopropane-1-carboxylate	52918-63-5	505.206
	Fenvalerate/ Esfenvalerate	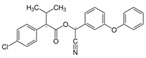	C_25_H_22_ClNO_3_	(*RS*)-*alpha*-Cyano-3-phenoxybenzyl (RS)-2-(4-chlorophenyl)-3-methylbutyrate	51630-58-1	419.91
	Cyfluthrin	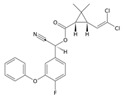	C_22_H_18_Cl_2_FNO_3_	(*R*)-Cyano(4-fluoro-3-phenoxyphenyl)methyl (1*R*,3*R*)-3-(2,2-dichloroethen-1-yl)-2,2-dimethylcyclopropane-1-carboxylate	68359-37-5	434.29
	Cyhalothrin	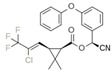	C_23_H_19_ClF_3_NO_3_	[cyano-(3-phenoxyphenyl)methyl] 3-[(Z)-2-chloro-3,3,3-trifluoroprop-1-enyl]-2,2-dimethylcyclopropane-1-carboxylate	91465-08-6	449.85
	Fenpropathrin	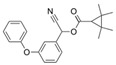	C_22_H_23_NO_3_	[Cyano-(3-phenoxyphenyl)methyl] 2,2,3,3-tetramethylcyclopropane-1-carboxylate	39515-41-8	349.430
	Flucythrinate	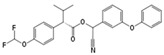	C_26_H_23_F_2_NO_4_	Cyano(3-phenoxyphenyl)methyl 2-[4-(difluoromethoxy)phenyl]-3-methylbutanoate	70124-77-5	451.470
	Fluvalinate	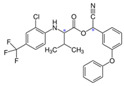	C_26_H_22_ClF_3_N_2_O_3_	[Cyano-(3-phenoxyphenyl)methyl] 2-[2-chloro-4-(trifluoromethyl)anilino]-3-methylbutanoate	69409-94-5	502.92
	Tralomethrin	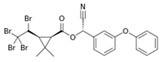	C_22_H_19_Br_4_NO_3_	(1*R*,3*S*)-2,2-Dimethyl-3-(1,2,2,2-tetrabromoethyl)-1-cyclopropanecarboxylic acid [(*S*)-cyano-[3-(phenoxy)phenyl]methyl] ester	66841-25-6	665.014
	Flumethrin	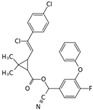	C_28_H_22_Cl_2_FNO_3_	Cyano(4-fluoro-3-phenoxyphenyl)methyl 3-[2-chloro-2-(4-chlorophenyl)vinyl]-2,2-dimethylcyclopropanecarboxylate	69770-45-2	510.39
	Imiprothrin	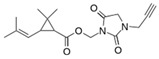	C_17_H_22_N2_O4_	[2,5-Dioxo-3-(prop-2-yn-1-yl)imidazolidin-1-yl]methyl 2,2-dimethyl-3-(2-methylprop-1-en-1-yl)cyclopropane-1-carboxylate	72963-72-5	318.373
	Flucythrinate	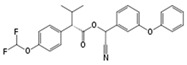	C_26_H_23_F_2_NO_4_	Cyano(3-phenoxyphenyl)methyl 2-[4-(difluoromethoxy)phenyl]-3-methylbutanoate	70124-77-5	451.470
	Cycloprothrin	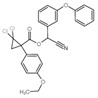	C_26_H_21_Cl_2_NO_4_	[cyano-(3-phenoxyphenyl)methyl] 2,2-dichloro-1-(4-ethoxyphenyl)cyclopropane-1-carboxylate	63935-38-6	482.4
Others	Etofenprox	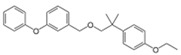	C_25_H_28_O_3_	1-{[2-(4-Ethoxyphenyl)-2-methylpropoxy]methyl}-3-phenoxybenzene	80844-07-1	376.496

**Table 2:** Biomarkers involved in the estrogenicity of pyrethroids.

**In vitro markers**							
**References**	**Pyrethroids**	**Targets**		**Samples**	**Dosages**	**Treatment Days**	**Transcriptional** **Changes of Biomarkers**
		**Gene**	**Protein**				**Upregulated (↑) or** **Downregulated (↓)**
[37]	Sumithrin or d-Phenothrin	pS2	-	MCF7 cells	10^−5^–10^−12^ M	3	pS2 (↑), ERα (↑), ERβ (↓)
	bioallethrine, cypermethrin, deltamethrin, fenvalerate, permethrin, and tetramethrin						No significant changes
[32]	Cypermethrin Permethrin	pS2 ERα	-	MCF-7	10^−5^–10^−9^ M	2	pS2 (↑), ERα (↑)
[25]	α-cypermethrin, β-cypermethrin	3-βHSD, 17-βHSD, CYP19, STAR, and CYP11B2	-	H295R	10^−5^–10^−7^ M	2	3-βHSD (↓), 17-βHSD (↑), CYP19 (↑), STAR (↑) CYP11B2 (↑)
	θ-cypermethrin						3-βHSD (↓)
**In vivo markers**							
**References**	**Pyrethroids**	**Targets**		**Samples**	**Dosages**	**Treatment days**	**Transcriptional** **changes of biomarkers**
		**Gene**	**Protein**				**Upregulated (↑) or** **Downregulated (↓)**
[27]	Bifenthrin	P450scc, StAR, PR, AREG, TGF-β1, C/EBP β, RUNX1, p21, cyclin E1, CYP19a1,SULT1E1, PTGS2, PGE_2_	-	rat ovarian granulosa cells	10^−6^–10^−8^ M	6 hrs after treatment with hCG (1 IU/mL)	PTGS2 (↓), PR (↓), SULT1E1 (↓), PGE_2_ (↓), P450scc (↓), StAR (↓) AREG (↓), TGF-β1 (↓) C/EBP β (↓), RUNX1 (↓) p21 (↓), cyclin E1 (↓), CYP19a1 (↓)
[30]	Tetramethrin	CaBP-9k	ERα, ERβ	uterus of immature rats	5–800 mg/kg S.C.	3	CaBP-9k (↓), ERα (↓) ERβ (no significant change)
[31]	Permethrin	CaBP-9k	-	uterus of immature rats	10 to 800 mg/kg S.C.	3	CaBP-9k (↑)
[37]	Sumithrin Tetramethrin	ERα, ERβ, and CaBP-9k	-	Female SD rat uteri	5–800 mg/kg/day S.C.	3	ERα (↑), ERβ (↑), CaBP-9k (↓)
[27]	Bifenthrin	P450scc, StAR, PR, AREG, TGF-β1, C/EBP β, RUNX1, p21, cyclin E1, CYP19a1, SULT1E1, PTGS2	-	gonadotropin-primed immature female rats	0.5–5 mg/kg I.P.	24	PTGS2 (↓), PR (↓), SULT1E1 (↓), PGE_2_ (↓), P450scc (↓), StAR (↓), AREG (↓), TGF-β1 (↓), C/EBP β (↓), RUNX1 (↓), p21 (↓), cyclin E1 (↓), CYP19a1 (↓)
[26]	Cypermethrin		Lactate dehydrogenase (LDH), 3-βHSD	Rat ovary	50 mg/kg, P.O.	14	LDH (↓), 3-βHSD (↓)

S.C., subcutaneous injection; I.P., intravenous injection; P.O.; oral administration.

**Table 3:** Estrogenic receptor binding of some pyrethroids.

References	Method	Pyrethroids	Agonistic	
			Doses (M)	IC50 (μM)
[40]	Fluorescence polarization method	d-trans-allethrin, cypermethrin, empenthrin, fenvalerate, imiprothrin, permethrin, d-phenothrin, and prallethrin	10^−5^ to 10^−8^	N
[39]	Competitive receptor binding using 3,6,7-[3H] estradiol with varying concentrations of radio-inert competitor	Permethrin		-
		Bioallethrin		-
			10^−4^ to 10^−12^	
		Fenvalerate		479
		Cypermethrin		562
		Permethrin, Deltamethrin		<67% of IC50
[37]	Radiolabeled estrogen [3H]estradiol	Sumithrin, BioAllethirn, Cypermethrin, Deltamethrin, Fenvalerate, Permethrin, Tetramethrin,	10^−4^ to 10^−14^	-

IC: inhibition concentration; N: negative.

**Table 4:** Results of the agonistic and antagonistic ER transactivation assay of some pyrethroids.

Reference	Pyrethroids	Cell Type	Agonistic			Antagonistic	
			RPCmax (%)	PC_20_ (M)	EC_50_ (M)	PC_20_ (M)	EC_50_ (M)
[40]	Cypermethrin,	Hela	N	-	-	-	
	Empenthrin						
	Permethrin, Prallethrin						
	Fenvalerate, Allethrin						
	Imiprothrin, d-phenothrin						
[49]	Fenvalerate	CHO-K1	50	3.70 × 10^−6^	-		
	Flucythrinate		31	5.70 × 10^−6^	-		
	Cyfluthrin		45	5.90 × 10^−6^	-	10^−10^	-
	Cypermethrin		28	8.10 × 10^−6^	-	10^−10^	-
	Permethrin		24	8.40 × 10^−6^	-		
					
	Deltamethrin		-	-	-		
	Cyhalothrin		-	-	-		
[48]		CV-1					
	Cypermethrin		-	4.14 × 10^−6^	-	N	-
	Deltamethrin		-	-	-	N	-
					
	Permethrin		-	8.10 × 10^−7^	-	>10^−5^	-
	Tetramethrin		-	-	-	N	-
	Cyhalothrin		-	3.34 × 10^−8^	-	N	-
	Cyfluthrin		-	-	-	1.36 × 10^−6^	-
	Fenvalerate		-	1.80 × 10^−6^	-	N	-
	Cycloprothrin		-	N	-	2.30 × 10^−8^	-
	Etofenprox		-	N	-	3.50 × 10^−8^	-
[41]	Cypermethrin	Hela	11.4	-	-	-	
	Fenvalerate		55	-	2.7 × 10^−6^		
	Permethrin		13	-	-		
[28]	Deltamethrin	MCF-7 (BUS)	N	-	-	-	
[47]	Permethrin	BG-1	N	-	-		-
	Bifenthrin		60	N	-		
[37]	Cypermethrin (pure)	Mouse Sertoli cells			0.001	-	
	Permethrin		-	-	9.30 × 10^−6^		
	Alpha				1.00 × 10^−5^		
	cypermethrin						
			
	Bifenthrin						
	Deltamethrin				N		
	Cyfluthrin		-	-			
	Taufluvalinate						
[45]	Fenvalerate	Ishikawa Var-1 Endometrial cancer cells	-		-		-
	Sumithrin			10^−5^			
	d-trans allethrin			10^−5^			
	permethrin						

PC: positive concentration; EC_50_: Half maximal effective concentration; RPCMax: maximum level of response induced by an E_2;_ N: negative.

**Table 5:** Comparative data on MCF7 cell proliferation after pyrethroid exposure.

References	Pyrethroids	Concentration (M)	LOEL, M
[51]	Sumithrin, Fenvalerate, d-trans Allethrin Permethrin	10^−4^–10^−9^	10^−5^
			10^−4^
			10^−5^
[28]	Deltamethrin	-	N
[39]	Fenvalerate	10^−6^–10^−11^	10^−6^
	Permethrin		10^−7^
	Cypermethrin		10^−8^
	Deltamethrin		10^−6^
[53]	Permethrin	10−4–10−6	10^−7^
	Cypermethrin		10−7
[37]	Sumithrin	10^−5^–10^−7^	10^−5^
[57]	1S-cis-bifenthrin, 1R-cis-bifenthrin	10^−5^–10^−7^	N
[32]	Permethrin, β-cypermethrin Metabolites (3-PBAlc, 3-PBAld, 3-PBAcid)	10^−5^–10^−9^	10^−9^
[55]	Lambda-cyhalothrin	10^−5^–10^−13^	10^−7^
[52]	Fenvalerate	10^−4^–10^−8^	10^−8^
[54]	Permethrin	10^−4^–10^−7^	10^−7^

LOEL; Lowest observed effect level; N = negative.

**Table 6:** In vivo data (Uterotrophic activity) of pyrethroids for the Integrated Approaches to Testing and Assessment.

References	Pyrethroids	Species	Routes	Treatment Days	Dosage (mg/kg)	LOEL (mg/kg)	Results
[71]	Cypermethrin	Albino rats	PO	30	5, 20	-	-
[72]		Albino rats	PO	28	50	50	P
[73]	Fenvalerate	Non/Pregnant rats	PO	120	40 and 40: 80	-	N
[74]		SD rats	PO	28	1.91, 9.55, 31.80	-	N
[67]		Pregnant wistar rats	PO	GD 12 to PND 12	40	-	N
[66]		IMT female rats	PO	3	0.4, 4, 8, 40	-	N
[65]		OVX female rats	PO	3	20, 40, 80	-	N
[75]	Deltamethrin	Albino pregnant rats	PO	Until GD 7	1, 2, 4	-	N
[76]		Albino pregnant rats	PO	7	1, 2, 4	-	-
[77]		Wistar rats	I.P.	14	2.5, 5, 10	-	-
[68]		Pregnant Wistar rats	PO	21	1, 2	-	-
[78]		IMT female rats	PO	3	2, 4	N	-
[65]	Esfenvalerate	OVX female rats	PO	3	5, 10, 20	N	-
[65]	Permethrin	OVX female rats	PO	3	37.5, 75, 150	N	-
[31]		IMT female rats	S.C.		10, 50, 100, 200, 800	800	P
[70]	Bifenthrin	SD rats	PO	3	1.47, 4.41, 13.23	13.23	P
[30]	Tetrametrin	IMT female rats	S.C.	3	5, 10, 50, 100, 200, 800	5	P
[36]	Lambda cyhalothrin	Wistar rats	PO	14	6.3, 11.33	6.3	P
[79]	d-Phenothrin	IMT female rats	PO	3	100, 300, 1000	-	N

OVX; ovariectomized, IMT; immature, GD; Gestational day, PND; Post-natal day, PO; oral administration, SC; subcutaneous injection, IP; intraperitoneal injection, LOEL; Lowest observed effect level, P; positive, N; negative.

**Table 7:** Summary of the estrogenic ITS of pyrethroids.

**Data-Rich**					
**Pyrethroids**	**MIE**	**KIE**			**Adverse Outcome**
	**Hormones/Enzymes**	**ER** **α** **Receptor Binding (IC_50_, μM)**	**ER Transcription** **(PC_20_, M)**	**MCF-7 Cell Proliferation** **(LOEL, M)**	**Uterotrophic Activity** **(LOEL; mg/kg)**
Cypermethrin	Upregulated pS2 gene and aromatase	562	8.1 × 10^−6^	10^−7^–10^−8^	50
Permethrin	-	N	N	N	800
Lambda cyhalothrin	Changes in Steroidogenesis	No data	3.34 × 10^−8^ *	10^−7^	6.3
Tetramethrin	Downregulation of CaBP-9k and ERα	N	N *	N	5
Sumithrin/ d-phenothrin	Downregulation of ERα	N	N	10^−5^	No effects
Deltamethrin	-	< 67%	10^−10^ (antagonistic)	10^−6^	No effects
		N	N	10^−6^ (Partial agonistic)	
		-	N	10^−6^ (Antagonistic)	
Fenvalerate	-	479	0.27 μM (PC_50_) *	10^−5^–10^−6^	No effects
**Data-Poor**					
**Pyrethroids**	**MIE**	**KIE**			**Adverse Outcome**
	**Hormones/Enzymes**	**ER** **α** **Receptor Binding (IC_50_, μM)**	**ER Transcription** **(PC_20_, M)**	**MCF-7 Cell Proliferation** **(LOEL, M)**	**Uterotrophic Activity** **(LOEL; mg/kg)**
Bifenthrin	-	-	-	N	13.23
Esfenvalerate	Inhibition of LH	-	-	-	N
Bioallethrin	-	N	-	N	-
Allethrin	-	-	N	10^−4^	-
Prallethrin	-	-	N	-	-
Imiprothrin	-	-	N	-	-
Flucythrinate	-	-	5.7 × 10^−6^	-	-
Cyfluthrin	-	-	5.9 × 10^−6^	-	-
Cycloprothrin	-	-	N	-	-
Empenthrin	-	-	N	-	-
Etofenprox	-	-	3.50 × 10^−8^ (antagonistic)	-	-

* Hela cells were used based on the OECD guidelines, N = negative, LOEL; Lowest observed effect level, PC: positive concentration; IC: inhibition concentration; RPCMax: maximum level of response induced by a test chemical.

## Data Availability

The data presented in this study are available on request from the corresponding author.
